# Diminished Pneumococcal-Specific CD4^+^ T-Cell Response is Associated With Increased Regulatory T Cells at Older Age

**DOI:** 10.3389/fragi.2021.746295

**Published:** 2021-11-03

**Authors:** Samantha W. J. He, Martijn D. B. van de Garde, Daan K. J. Pieren, Martien C. M. Poelen, Franziska Voß, Mohammed R. Abdullah, Sven Hammerschmidt, Cécile A. C. M. van Els

**Affiliations:** ^1^ Centre for Infectious Disease Control, National Institute for Public Health and the Environment, Bilthoven, Netherlands; ^2^ Department of Molecular Genetics and Infection Biology, Interfaculty Institute of Genetics and Functional Genomics, Center for Functional Genomics of Microbes, University of Greifswald, Greifswald, Germany

**Keywords:** *Streptococcus pneumoniae*, CD4^+^ T cells, aging, tregs, infection, immunosenescence, pneumococcal proteins, proinflammatory cytokines

## Abstract

Respiratory infection caused by *Streptococcus pneumoniae* is a leading cause of morbidity and mortality in older adults. Acquired CD4^+^ T cell mechanism are essential for the protection against colonization and subsequent development of infections by *S. pneumoniae*. In this study, we hypothesized that age-related changes within the CD4^+^ T-cell population compromise CD4^+^ T-cell specific responses to *S. pneumoniae*, thereby contributing to increased susceptibility at older age. To this end, we interrogated the CD4^+^ T-cell response against the immunogenic pneumococcal protein AliB, part of the unique oligopeptide ABC transporter system responsible for the uptake of nutrients for the bacterium and crucial for the development of pneumococcal meningitis, in healthy young and older adults. Specifically, proliferation of CD4^+^ T cells as well as concomitant cytokine profiles and phenotypic markers implied in immunosenescence were studied. Older adults showed decreased AliB-induced CD4^+^ T-cell proliferation that is associated with an increased frequency of regulatory T cells and lower levels of active CD25^+^CD127^+^CTLA-4^−^TIGIT^-^CD4^+^T cells. Additionally, levels of pro-inflammatory cytokines IFNy and IL-17F were decreased at older age. Our findings indicate that key features of a pneumococcal-specific CD4^+^ T-cell immune response are altered at older age, which may contribute to enhanced susceptibility for pneumococcal infections.

## Introduction

*Streptococcus pneumoniae* is a leading cause of respiratory infections in adults above 65 years of age, which is associated with substantial morbidity and mortality (Vila-Corcoles and Ochoa-Gondar, 2013; Drijkoningen and Rohde, 2014). The Gram-positive bacterium resides as a commensal in the human nasopharynx, with highest carriage rates in children under 5 years old, but can become invasive depending on microbial, environmental and host factors (Hussain et al., 2005; Abdullahi et al., 2012; Weiser et al., 2018).

The natural incidence of invasive pneumococcal disease (IPD) describes a typical U-shape throughout life, with reduced rates as children become older and a sharp increase at older age (Jansen et al., 2009). These shifts in susceptibility are most likely explained by alterations in the acquired host immune mechanisms with age.

Pneumococcal protein-specific CD4^+^ T cells are considered key players in the control of pneumococcal infections. Stimulation of CD4^+^ T cells with pneumococcal strains induces proliferation and cytokine secretion by Th1, Th2 and Th17 memory CD4^+^ T cells, with predominant pro-inflammatory Th1 and Th17 responses (Engen et al., 2014). IL-17, in particular, was shown to enhance the recruitment of phagocytes to the site of invasion and boost their phagocytic abilities, leading to rapid and effective clearance of *S. pneumoniae* via opsonophagocytosis (Lu et al., 2008; Zhang et al., 2009; Wright et al., 2013). The induced effector CD4^+^ T-cell response is also modulated by pneumococcal-specific regulatory T cells (Tregs) to prevent excessive tissue damage and subsequent severe infection due to compromised tissue integrity and barriers (Pido-Lopez et al., 2011; Neill et al., 2012). Both low CD4^+^ T-cell effector responses and increased numbers of Tregs are associated with positive pneumococcal carriage in children and young adults (Zhang et al., 2007; Zhang et al., 2011; Jiang et al., 2015; Mubarak et al., 2016), highlighting the importance of these responses in regulating colonization, a pre-requisite towards disease. However, less is known about alterations in pneumococcal-specific CD4^+^ T cell immunity at older age.

Deterioration of immune responses with age is a well-known phenomenon, termed immunosenescence, that reduces the ability of older adults to respond to infections and vaccines (Ginaldi et al., 2001). Decreased CD4^+^ T-cell proliferation is one of the hallmarks of immunosenescence that affects the efficiency of the CD4^+^ T-cell response, as observed in aged mice and humans (Wikby et al., 1998; Aiello et al., 2019; Pieren et al., 2019). In addition, constitutive expression of inhibitory co-receptors including Programmed cell-death (PD-1) and Cytotoxic T-lymphocyte-associated antigen 4 (CTLA-4) on T cells (Leng et al., 2002; Channappanavar et al., 2009) as well as accumulation of Tregs, observed in aged mice (Nishioka et al., 2006; Sharma et al., 2006; Lages et al., 2008), could counteract and imbalance the stimulatory signal of T cells upon activation, thereby resulting in reduced CD4^+^ T cell response at older age. To explore whether human anti-pneumococcal CD4^+^ T cell immunity is negatively impacted at older age, we examined the presence of these immunosenescent features on CD4^+^ T cells of young and older adults using an important class of pneumococcal proteins.

AmiA, AliA, AliB, AliC and AliD are substrate binding lipoproteins, showing approximately 60% sequence homology (Alloing et al., 1994; Hathaway et al., 2004). AmiA, AliA and AliB are known to induce antibody responses in humans (Croucher et al., 2017). The proteins are part of the unique Ami-AliA/B oligopeptide ABC transporter system of *S. pneumoniae* and are essential for the uptake of nutrients and environmental signals by the bacteria (Nasher et al., 2017; Nasher et al., 2018). Furthermore, AmiA and AliA are involved in the colonization of the nasopharynx (Kerr et al., 2004), while AliB is essential for the development of pneumococcal meningitis (Schmidt et al., 2019). AliC and AliD are AliB-like homologues only expressed by non-encapsulated *S. pneumoniae* (NESP) (Hathaway et al., 2004; Keller et al., 2016).

In our study of the CD4^+^ T-cell response against the broadly recognized AliB protein, evidence was provided suggesting that older adults experience diminished pneumococcal-specific CD4^+^ T-cell responses associated with increased Treg-mediated regulation. This could contribute to increased susceptibility for IPD at older age.

## Materials and Methods

### Ethics Statement and Study Population

Buffy coats were obtained from healthy adult blood donors in the age range of 20–75 years old with written consent in accordance with local protocols for blood products destined “not for transfusions” (Sanquin Blood Supply, Netherlands). In total, thirty-six healthy adults (20 females and 16 males) were included in this study. Twenty-four donors were sub-selected in two age cohorts of 20–35 year old (young adults, *n* = 12) and 60–75 year old (older adults, *n* = 12), respectively, to study aging of pneumococcal-specific CD4^+^ T-cell responses in detail.

### PBMC Isolation

Peripheral Blood Mononuclear Cells (PBMCs) were isolated from the buffy coats via Lymphoprep (Axis-shield) density gradient centrifugation using standard procedures and frozen in 10% DMSO. Samples were kept at −80°C overnight and then stored at −135°C until use.

### Pneumococcal Antigens

Pneumococcal protein AliB with N-terminal His_6_-tag was recombinantly produced and purified as previously described (Schmidt et al., 2019). The other pneumococcal proteins AmiA, AliA, AliC and AliD were generated by cloning amplified target genes *amiA* (sp_1891, nt 122-2032), *aliA* (spd_0334, nt 85-1980), *aliC* (nt 73-1965), and *aliD* (nt 82-1965), without their signal sequences, into the pTP1 expression vector (Saleh et al., 2013). PCR reactions were performed using *S. pneumoniae* TIGR4 (*amiA*), D39 (*aliA*), or MNZ41 (*aliC*, *aliD*) chromosomal DNA as a template and the primer pairs listed in Table 1. The primers contained restriction sites (*Nhe*I/*Sac*I, *Spe*I/*Hin*dIII), which were used to ligate the fragments into the similarly digested expression vector. Resulting plasmids were transformed into competent *E. coli* BL21 (DE3) for heterologous protein expression. Protein expression and purification of N-terminal His_6_-tag fusion proteins were performed as previously described (Saleh et al., 2013). Purity of the proteins was subsequently analyzed by SDS-PAGE followed by silver staining or immunoblotting with anti-Penta-His-tag mouse antibody (Qiagen) (Voss et al., 2018).

**TABLE 1:** Primer list.

Primer name	Primer No.	Restriction site	Sequence (5′-3′)
AmiA_F	1562	*Nhe*I	GCG​CGCGCT​AGCAGT​TCT​TCA​AAA​TCA​TCT​GAT​TC
AmiA_R	1563	*Sa*cI	GCG​CGCGAG​CTCTTA​CTT​CAC​ATG​ACT​TGC​CAA​TTC
AliA_F	1931	*Nhe*I	AAT​TGCT​AGCTCT​GGA​TCA​GGT​TCA​AGC
AliA_R	1932	*Sa*cI	GCG​CGAG​CTCATT​TCA​CAT​GTT​TTG​C
AliC_F	1921	*Nhe*I	GCG​CGCGCT​AGCAAA​AGT​GAA​AAG​AAT​GC
AliC_R	1922	*Sa*cI	GCG​CGCGAG​CTCATT​TTA​TGT​GCT​TTT​C
AliD_F	1923	*Spe*I	GCG​CGCACT​AGTTCA​GAT​ACA​AAA​ACT​TAC
AliD_R	1924	*Hin*dIII	GCG​CGCAAG​CTTATT​TAA​CAT​GTT​TTT​CTG​C

### Thymidine Incorporation Assay

PBMCs from donors were seeded at a density of 1.5 × 10^5^ cells/well in AIM-V medium (Gibco, Thermo Fisher Scientific) containing 2% human AB serum (Sigma-Aldrich) in 96-well U-bottom plates. Cells were stimulated with medium control (mock), AmiA, AliA, AliB, AliC or AliD at 1 μg/ml, in replicate wells per condition for 6 days at 37°C with 5% CO_2_ in the absence or presence of anti-MHC class II blocking antibodies consisting of a cocktail of anti-HLA-DR (B8.11-2; in-house produced), anti-HLA-DQ (SPV-L3; in-house produced) and anti-HLA-DP (B7/21; Leinco Technologies) monoclonal antibodies, as indicated. Tritium thymidine (18 kBq/well) was added to the wells on day six before overnight incubation at 37°C with 5% CO_2_. Cells were then harvested on a filter and incorporated label was determined as counts per minute (cpm) using a MicroBeta Counter (Perkin Elmer). Tritium thymidine based stimulation indices (SI) were calculated by dividing the mean cpm of the replicate stimulated wells, by the mean cpm of the quadruplicate medium control wells. SI’s > 1.7 were considered positive (van de Garde et al., 2019).

### CellTrace-Based CD4^+^ T-Cell Proliferation Assay

PBMCs from donors were rapidly thawed at 37°C and labeled with CellTrace™ Violet proliferation dye according to manufacturer’s protocol (Gibco, Thermo Fisher Scientific). CellTrace™ Violet-labelled cells were seeded at a density of 1 × 10^6^ cells/well in a 48-well flat-bottom plate in AIM-V medium with 2% AB serum and stimulated with either mock, AmiA, AliA, AliB, AliC or AliD at 1 μg/ml, individually, in replicate wells per condition at 37°C with 5% CO_2_ for 5 and 7 days, in separate plates per timepoint. Cells were harvested from both day 5 and day 7 cultures, after which replicates were pooled, washed and used for flow-cytometric analysis. Based on optimization assays, supernatants for cytokine analysis were collected from the day seven cultures, pooled per stimulus and stored at −80°C until use.

### Flow-Cytometric Analysis

Before and after *in vitro* culturing, CellTrace™ Violet-labeled cells were stained with the following fluorochrome-labeled antibodies at 4°C: CD3(SK7)-Alexa700, CD4(OKT-04)-BV711, CD8 (RPA-T8)-BV785, CD45RO(UCHL1)-PerCP-Cy5.5, FoxP3 (295D)-Alexa647, PD-1 (EH12.2H7)-BV605, CD127 (A019D5)-BV650, CTLA-4 (L3D10)-PE, Helios (22F6)-PE-Cy7, TIGIT (MBSA4)-PE/efluor-610 (ThermoFisher), CD27 (L128)-BUV395 (BD), CD25 (2A3)-BUV737 (BD) and Zombie NIR™ Fixable Viability kit (APC-Cy7, Biolegend). All antibodies are from Biolegend, unless stated otherwise. Acquisition was performed on BD LSR Fortessa X-20. Data was analyzed via FlowJo (Treestar). Frequencies of proliferated antigen-specific CD4^+^ T cells were determined by analyzing sequential halving of the Cell trace Violet fluorescence intensity within the CD4^+^ T cell gate (gating strategy in Supplementary Figure S1).

### Dimensionality Reduced Analysis

Dimensionality reduction (viSNE) analysis of flow cytometry data was performed in Cytobank (www.Cytobank.org) (Amir et al., 2013). All events within pre-gated CD4^+^ T-cell or proliferated CD4^+^ T-cell gates were concatenated in Flowjo for each age group per culture condition and uploaded to Cytobank. viSNE clustering was performed on equal samples of randomly selected 5 × 10^5^ cells from each age group based on expression of FoxP3, Helios, TIGIT, CTLA-4, PD-1, CD25 and CD127 expressions. Marker expression was represented as ArcSinh5-transformed medians within heatmaps generated via viSNE analysis.

### Cytokine Measurement

Levels of Th cell cytokines were measured in day 7 culture supernatants, with Bead-based LEGENDplex™ Human TH cytokines (13-plex) kit (Biolegend) according to the manufacturer’s instructions. Acquisition was performed on BD FACSCANTO™ II and data were analyzed with LEGENDplex™ Data Analysis Software. Levels of cytokines are expressed in pg/ml.

### Statistical Analyses

Statistical analysis was performed using GraphPad Prism 7 software (La Jolla, CA, United States). Significant differences between young and older adults were determined by non-parametric Mann-Whitney *U* test or parametric Two-way ANOVA. Correlations were tested with Spearman’s rank correlation coefficient. For all analyses, *p*-values < 0.05 were considered statistically significant.

## Results

### Pneumococcal Substrate-Binding Lipoproteins Induce CD4^+^ T-Cell Proliferation

Whilst AmiA, AliA and AliB are shown to be able to induce sustained antibody responses in humans (Croucher et al., 2017), it is not yet known whether this class of substrate-binding lipoproteins, including AliC and AliD, is able to elicit memory CD4^+^ T-cell responses. To investigate this, we first screened PBMCs from randomly selected healthy blood donors for recognition of AmiA, AliA, AliB, AliC and AliD in a thymidine incorporation assay. Results indicated that all five proteins were capable of inducing proliferative responses in PBMCs from healthy donors of random age, based on the calculated SI (Supplementary Figure S2).

**FIGURE 1:**
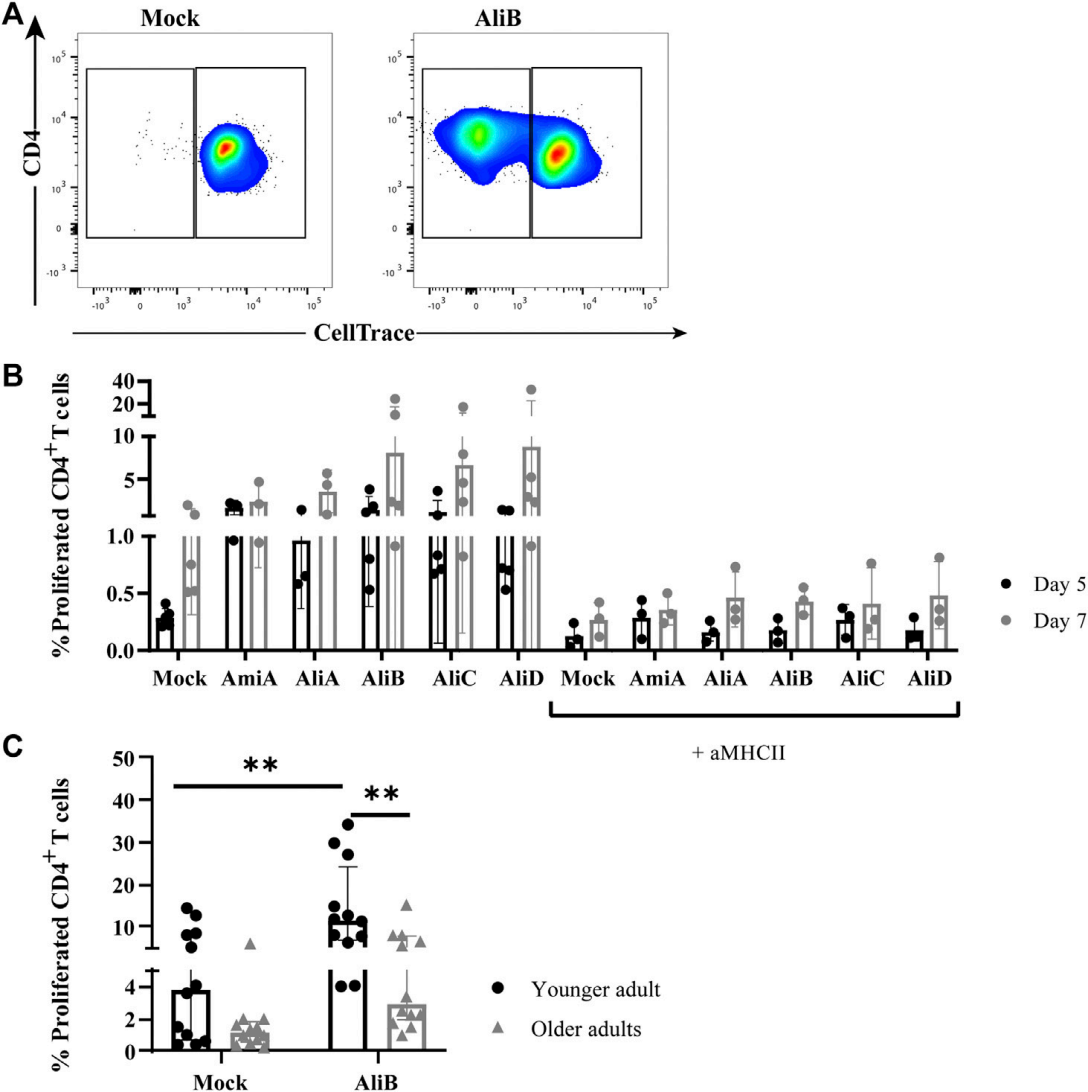
CD4^+^ T-cell immunogenicity of pneumococcal substrate-binding lipoproteins. **(A)** Example of gating strategy for proliferated (CellTrace^dim^) CD4^+^ T cells post 7 days stimulation with mock or AliB protein, as indicated (see general gating strategy of CD4^+^ T cells in Supplementary Figure S1). **(B)** Proliferation of PBMCs (*n* = 5 donors) stimulated with mock or five pneumococcal substrate-binding lipoproteins for five (black circles) and 7 days (grey circles) in the absence or presence of anti-MCH class II (aMHCII) blocking antibodies on both timepoints, as indicated. Bars represent mean with SD. **(C)** Frequency of proliferated CD4^+^ T cells in young (20–35 years old *n* = 12, black circles) and older adults (60–75 years old, *n* = 12, grey triangles) after CellTrace labeling and 7 days AliB stimulation of PBMCs. Bars represent median with interquartile range. Statistical significance is calculated with Mann Whitney *U*-test (** = *p* < 0.01).

To investigate whether the observed proliferation was CD4^+^ T-cell mediated, PBMCs from additional donors were labeled with CellTrace Violet and subsequently stimulated with all five pneumococcal proteins or mock. CellTrace diminution of CD4^+^ T cells was measured after five- and seven-day protein exposure, respectively, in the absence and presence of anti-MHC class II blocking antibodies. Depending on the donor, background CellTrace diminution of CD4^+^ T cells in mock-stimulated conditions could be observed. All five substrate-binding lipoproteins were CD4^+^ T-cell immunogenic as shown by the generation of a CellTrace^dim^ CD4^+^ T-cell population after protein stimulation (dot plot shown for AliB, Figure 1A) on day five that further expanded to day seven (Figure 1B). The presence of anti-MHC class II blocking antibodies fully inhibited the protein-specific responses to base-line levels of mock-stimulated cells on day five and day seven. This inhibition supports the notion that proliferation of the pneumococcal substrate-binding lipoprotein-specific CD4^+^ T cells involves recognition of cognate epitopes generated after protein processing and presentation in the context of MHC class II molecules by antigen presenting cells. Together these data establish that CD4^+^ T cell memory responses to AmiA, AliA, AliB, AliC and AliD are being acquired in adult donors, likely through pneumococcal carriage episodes throughout life, which are known to be immunizing (Malley et al., 2005; Ferreira et al., 2013; Wright et al., 2013; Wilson et al., 2015).

### No Phenotypical Differences in the *ex vivo* Naïve and Memory CD4^+^ T-Cell Subsets at Older Age

**FIGURE 2:**
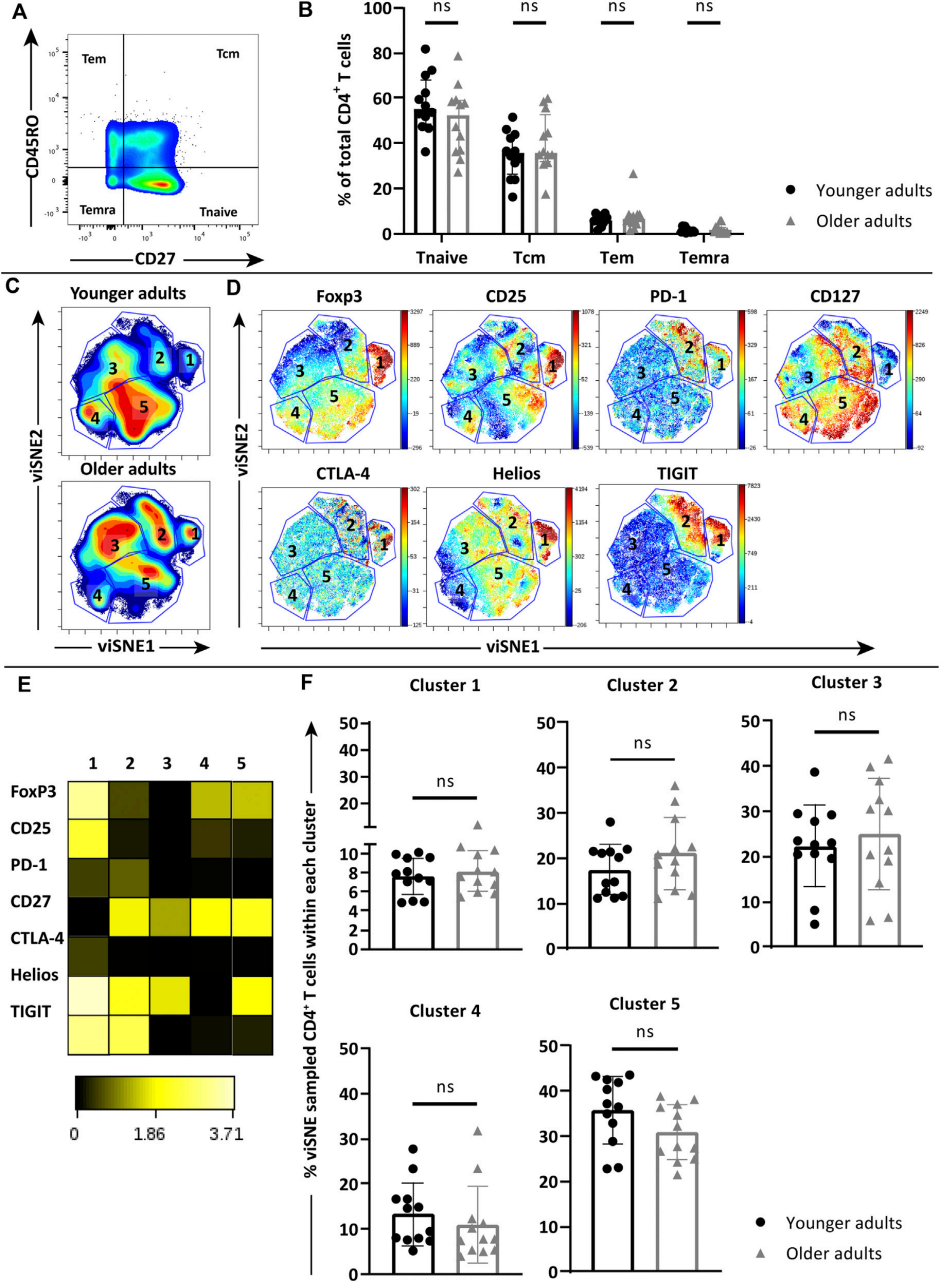
Phenotype analysis of *ex vivo* naïve and memory CD4^+^ T-cell subsets in young and older cohorts. **(A)** Gating of naïve (Tnaive), central memory (Tcm), effector memory (Tem) and terminally differentiated (TEMRA) CD4^+^ T cells based on CD45RO and CD27 expression. **(B)** Frequency of naive and memory subsets within *ex vivo* CD4^+^ T-cell pools of young (*n* = 12, black circles) and older adults (*n* = 12, grey triangles). **(C)** Density plots of pooled *ex vivo* CD4^+^ T cells of young (*n* = 12) and older adults (*n* = 12) clustered by viSNE based on the expression of FoxP3, CD25, PD-1, CD127, CTLA-4, Helios, and TIGIT. Five clusters were manually identified. **(D)** Fingerprint color dot plots showing the location and intensities of each marker within the identified clusters. **(E)** Heatmap depicts the ArcSinh5-transformed median expression of markers within each cluster. **(F)** Frequency of identified clusters within the total viSNE for each individual young (black circles) and older (grey triangles) donor. Bars represent median with interquartile range. Statistics is calculated with Two-way ANOVA in panel B and Mann Whitney *U*-test for panel F, ns = not significant.

Next we selected a panel of young (age 20–35 years old, *n* = 12) and older adult donors (age 60–75 years old, *n* = 12) to study whether features of immunosenescence negatively impact the observed anti-pneumococcal CD4^+^ T cell responses at older age. We first assessed whether age-dependent differences existed between the major CD4^+^ T-cell subsets of the young and older cohorts at baseline. Distribution of CD4^+^ T-cell memory subsets was compared between *ex vivo* PBMCs from the young (age 20–35 years old, *n* = 12) and older adults (age 60–75 years old, *n* = 12) based on expression of CD45RO and CD27 (Figure 2A). Comparable levels of naïve (Tnaive, CD45RO^−^CD27^+^), central memory (Tcm, CD45RO^+^CD27^+^), effector memory (Tem, CD45RO^+^CD27^−^) and terminally differentiated (Temra, CD45RO^−^CD27^−^) CD4^+^ T cells were found between the two age groups (Figure 2B).

We then investigated the *ex vivo* expression of co-inhibitory receptors PD-1 and CTLA-4, Treg-associated markers FoxP3, TIGIT and Helios, implied in immunosenescence of CD4^+^ T-cell responses, as well as activation marker CD25 (IL-2α receptor) and CD127 (IL-7α receptor) on the overall CD4^+^ T cell pools of young and older adults. Expression data were pooled per age group and subsequently clustered based on Median Fluorescence Intensity (MFI) of each marker by viSNE analysis. Five major clusters were manually identified in the pooled CD4^+^ T cells from the two age groups based on the densities (Figure 2C). Fingerprint expression plots (Figure 2D) showed clear clustering of FoxP3-expressing CD4^+^ T cells within cluster 1, which together with cluster 2 also contained CD4^+^ T cells with relatively higher expression of PD-1 and CTLA-4. Further examination of combined marker expression patterns within each cluster via heatmap (Figure 2E) showed that the CD4^+^ T cells in cluster 1 also expressed relatively high CD25, Helios and TIGIT with low expression of CD127, which together with the expression of FoxP3 suggest that cluster 1 consist of activated Tregs. However, comparison of the percentages of CD4^+^ T cells in each of the five clusters within the total viSNE of individual young and aged donors revealed no statistically significant differences (Figure 2F), indicating that the studied CD4^+^ T-cell phenotypes were similar at baseline between our age cohorts.

### Older Adults Show Altered CD4^+^ T-Cell Functional Responses to AliB Stimulation

To elucidate whether functional CD4^+^ T cell responses against the pneumococcal protein were impacted by age, we selected AliB as the prototype antigen as it evoked high CD4^+^ T-cell proliferative responses (Figure 1B) and was implied in pneumococcal disease (Schmidt et al., 2019).

CD4^+^ T-cell proliferation in the younger and older adult cohorts were compared after 7 days stimulation with AliB via CellTrace. Significantly decreased percentages of CellTrace^dim^ CD4^+^ T cells were observed in older adults compared to the younger cohort (median of 3.18 vs. 9.66%, respectively, *p* = 0.0024) (Figure 1C).

**FIGURE 3:**
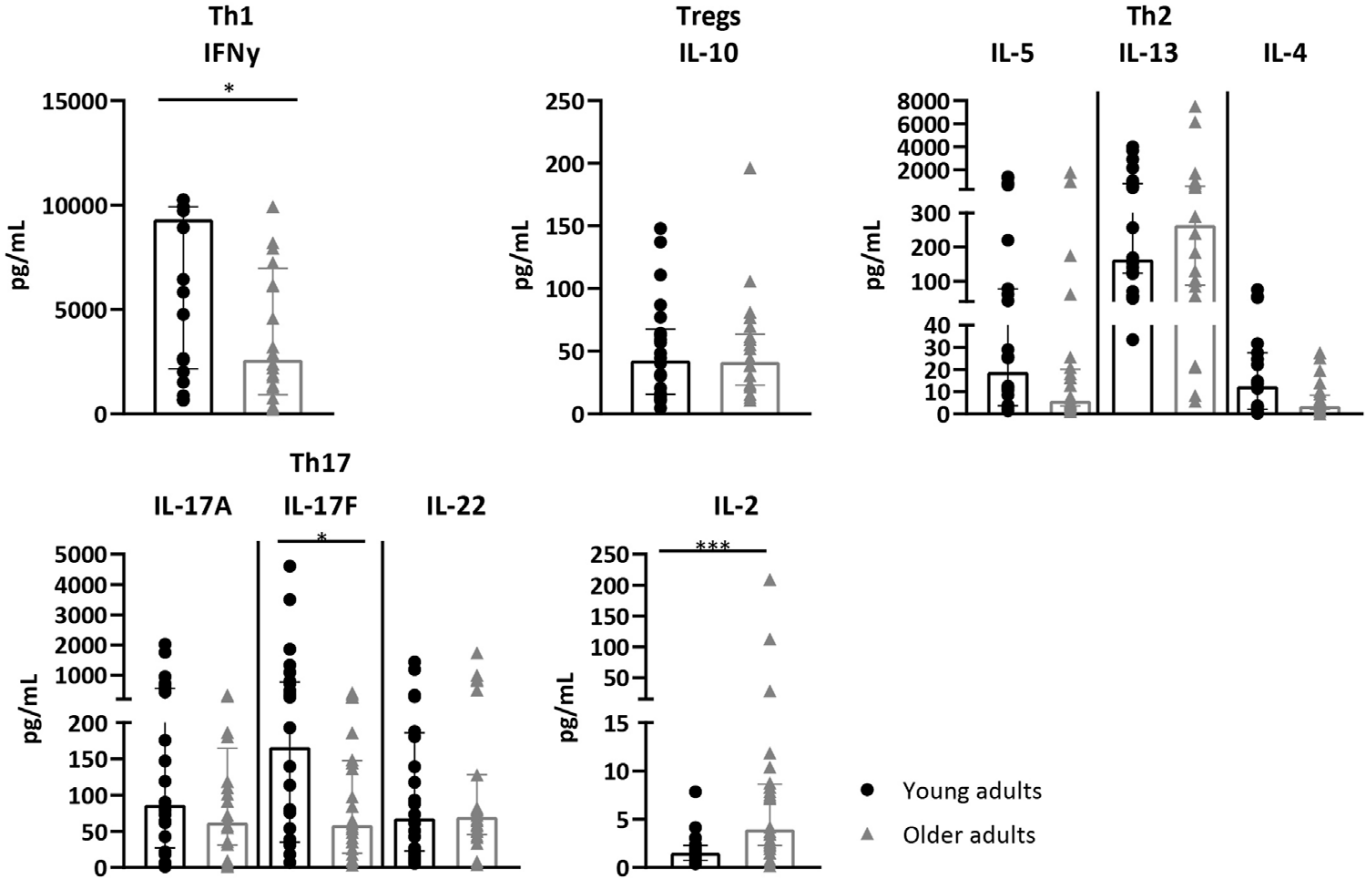
Cytokine profiles after AliB stimulation in young and older adults. Bar graphs show the levels of Th1, Th17, Th2, IL-10 and IL-2 cytokines, as indicated, measured in the supernatant of PBMCs of young (*n* = 12, black circles) and older (*n* = 12, grey triangles) adults after 7 days culture with AliB. Bars represent the median with interquartile range per age group. Statistical significance is calculated with Mann Whitney *U*-test (* = *p* < 0.05, *** = *p* < 0.001).

Next, we investigated whether reduced CD4^+^ T-cell proliferation in older adults was accompanied by differential Th-and Treg cytokine profiles compared to younger adults. Levels of IL-2, IL-9, Th1 (IFNy and TNFα), Th2 (IL-4, IL-5 and IL-13), Th17 (IL-17A, IL-17F, IL-21 and IL-22) and Treg-associated cytokines (IL-10) were measured in the supernatant after 7 days AliB stimulation. The levels of IFNy was significantly lower in older adults, whereas no differences in TNFα, IL-4, IL-5, IL-13, IL-10 and IL-9 were observed (Figure 3 + Supplementary Figure S3). Older adult PBMCs also showed significant lower levels of IL-17F and demonstrate a trend towards lower IL-17A and IL-22 production (Figure 3).

Interestingly, we also detected significantly increased levels of IL-2 in older adults after 7 days AliB stimulation. To investigate whether this was due to less efficient IL-2 consumption, expression of IL-2 receptor α-subunit, CD25, on proliferated CD4^+^ T cells was compared between young and older adults. Frequencies and MFI of CD25 were comparable between the two age cohorts (Supplementary Figure S4), indicating that higher IL-2 supernatant levels at older age may not be explained by altered CD25 expression.

Together, the decreased AliB-specific CD4^+^ T cell proliferation and Th1-Th17 cytokine profiles indicate an impaired functional response towards pneumococcal protein stimulation at older age.

### Altered Cluster Composition of Proliferated CD4^+^ T Cells at Older Age

**FIGURE 4:**
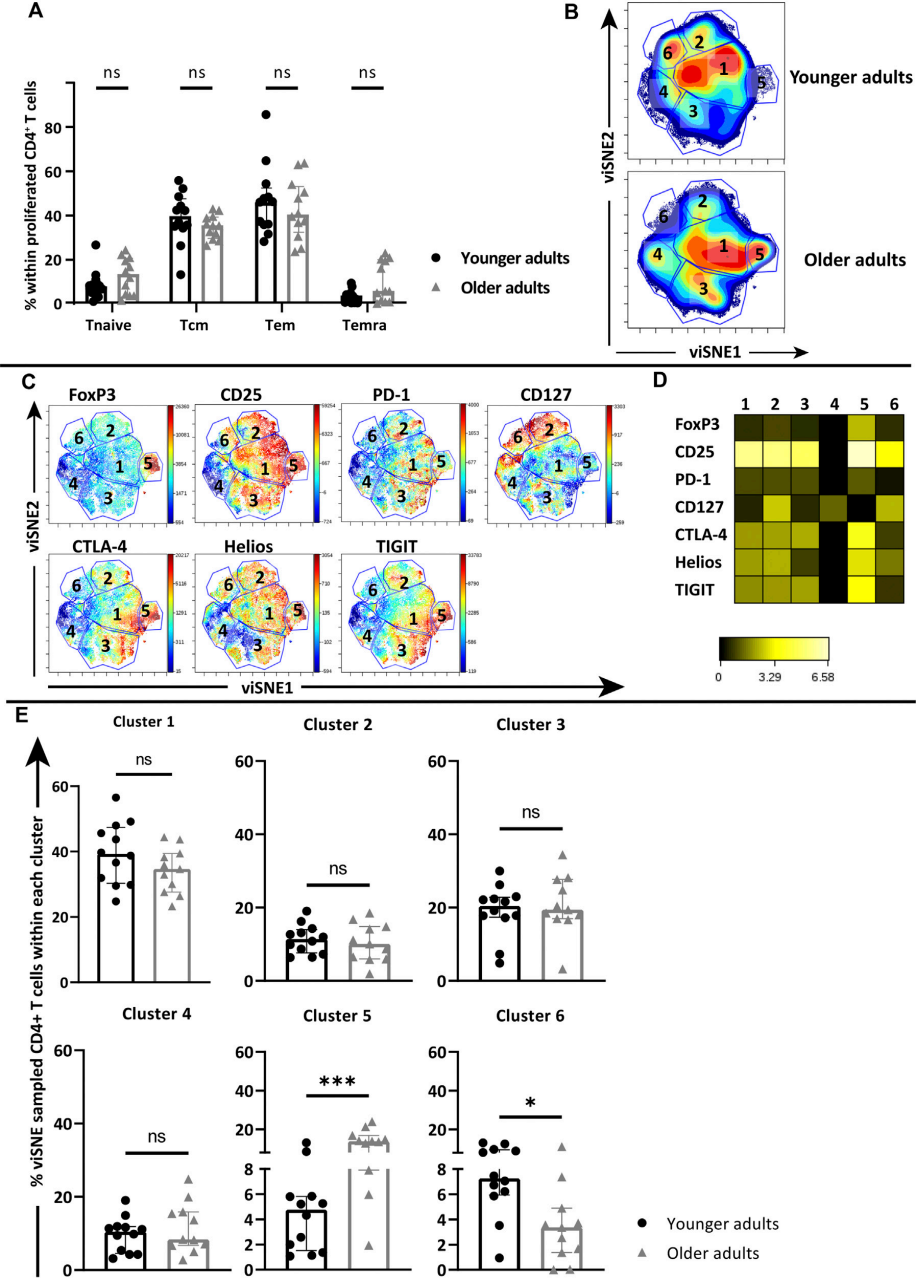
Cluster composition of proliferated CD4^+^ T cells in young and older cohorts. PBMCs of young and older adults were stimulated with AliB for 7 days. **(A)** Frequency of naive and memory subsets within proliferated CD4^+^ T-cell pools of young (*n* = 12, black circles) and older (*n* = 12, grey triangles) adults post stimulation. **(B)** Density plots of pooled proliferated CD4^+^ T cells from young and older adults clustered by viSNE based on the expression of FoxP3, CD25, PD-1, CD127, CTLA-4, Helios and TIGIT. Six main clusters were manually identified. **(C)** Fingerprint color dot plots show the location and intensities of each marker within the identified clusters. **(D)** Heatmap depicts the ArcSinh5-transformed median expression of markers within each cluster. **(F)** Bar graphs show the frequency of identified clusters within the total viSNE for each individual young (black circles) and older (grey triangles) donor. Bars represent median with interquartile range per age group. Statistical significance is calculated with Mann Whitney *U*-test (* = *p* < 0.05, ** = *p* < 0.01, *** = *p* < 0.001).

Next, we addressed the phenotypes within proliferated CD4^+^ T cells to see whether age-related differences were present after 7 days AliB stimulation that may explain the reduced proliferation and altered cytokine profile at older age. The proliferated CD4^+^ T cell populations from young and older adults did not differ in their distribution of Tnaïve, Tcm, Tem and Temra CD4^+^ T cell memory subsets, and both mainly comprised of Tcm and Tem cells (Figure 4A). To explore whether functional differences between the AliB-responding CD4^+^ T cells of young and older adults could be related to a differential expression of functionality markers, expression of PD-1, CTLA-4, FoxP3, TIGIT, Helios, CD25 and CD127 on pooled proliferated CD4^+^ T cells from each age group were analysed via viSNE. A total of 5 × 10^5^ proliferated CD4^+^ T cells were randomly sampled by viSNE from the pooled proliferated CD4^+^ T cells of young (*n* = 12) and older adults (*n* = 11, one donor excluded due to very low numbers of proliferated CD4^+^ T cells). After viSNE clustering, six major clusters were manually identified based on the density plots (Figure 4B). Both expression plots of individual markers (Figure 4C) and heatmap (Figure 4D) showed that FoxP3 expression is mainly restricted to cluster 5, whilst CD127 seemed to be associated with clusters 2, 4 and 6. Comparison of cluster frequencies in individual donors further showed that cluster 5 was significantly increased in older adults (median of 16.86 vs. 4.76%, respectively; *p* = 0.0007) (Figure 4E), whilst cluster 6 was significantly decreased compared to the younger age group (median of 3.36 vs. 7.25%; *p* = 0.012) (Figure 4E).

### Higher Frequencies of Tregs Associated With Decreased CD4^+^ T-Cell Proliferation in Older Adults After AliB Stimulation

Further examination of marker expression within cluster 5, via expression plot (Figure 4C) and heatmap (Figure 4D), revealed that the high FoxP3-expressing CD4^+^ T cells within this cluster also had relatively high expression of TIGIT and Helios, indicating that this enhanced cluster at older age consisted of activated Tregs (Figure 4D).

**FIGURE 5:**
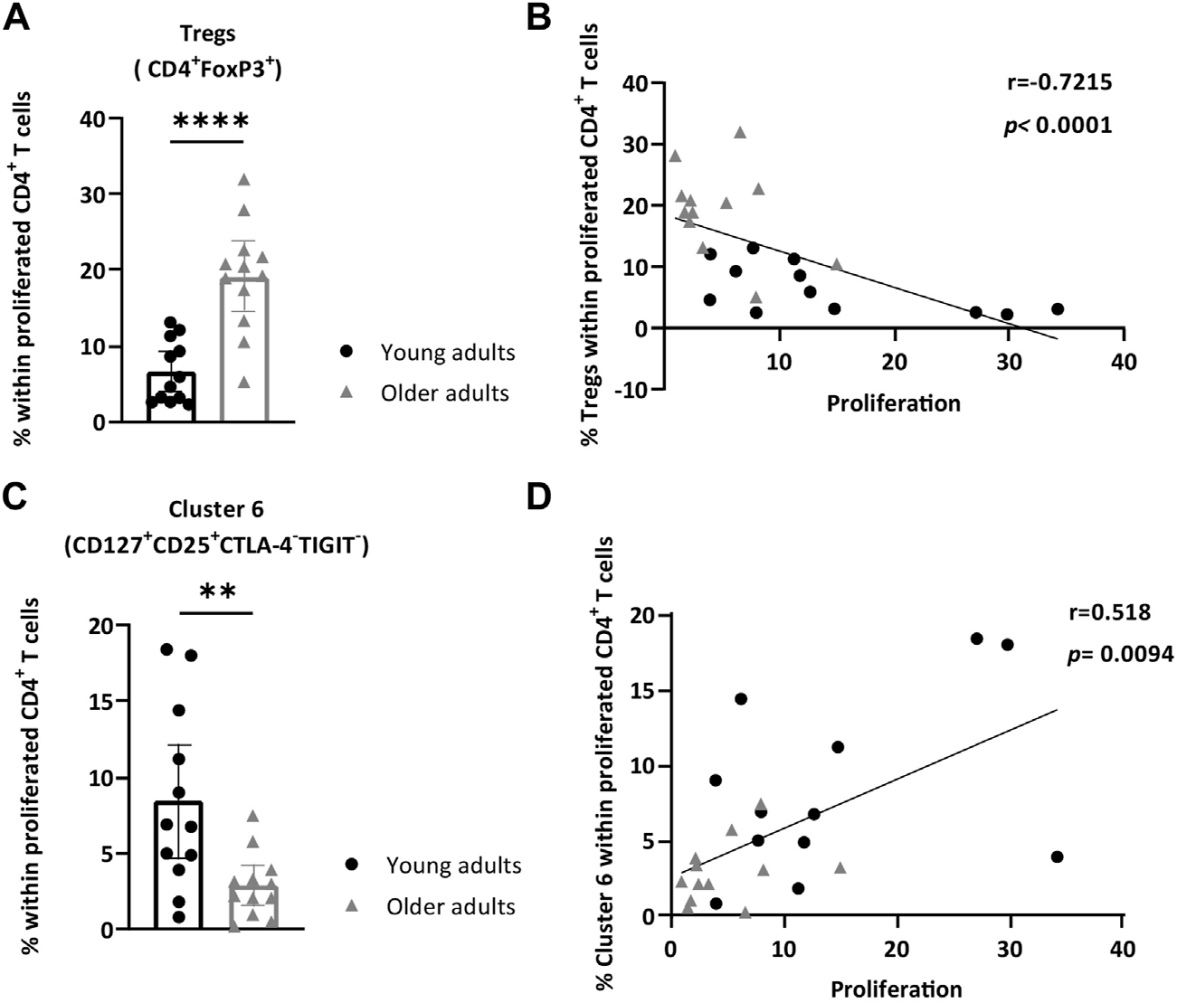
Increased frequency of Tregs and lowered levels of CD25^+^CD127^+^CTLA-4^-^TIGIT^-^ cells within responding CD4^+^ T cells are associated with low CD4^+^ T cell proliferation. PBMCs of young and older adults were stimulated with AliB for 7 days. **(A)** Bar graph depicts the frequencies of manually gated Tregs (FoxP3^+^CD4^+^ T cells) within proliferated CD4^+^ T cells in young (*n* = 12, black circles) and older (*n* = 12, grey triangles) adults. **(B)** Relationship between the frequency of Tregs in the responding CD4^+^ T cell fraction and frequency of proliferated CD4^+^ T cells (CellTrace^dim^) (*p* and *r*) was assessed by Spearman’s test. **(C)** Bar graph depicts the frequency of manually gated cluster 6 (CD25^+^CD127^+^CTLA-4^−^TIGIT^-^) within proliferated CD4^+^ T cells young (*n* = 12, black circles) and older (*n* = 12, grey triangles) adults. **(D)** Relationship between the frequency of cluster 6 in the responding CD4^+^ T cell fraction and proliferated CD4^+^ T cells is assessed by Spearman’s test (*p* and *r*). Bars represent median with interquartile range per age group. Statistical significance is calculated with Mann Whitney *U*-test (* = *p* < 0.05, ** = *p* < 0.01, *** = *p* < 0.001).

To assess whether the observed difference was applicable to the total proliferated CD4^+^ T-cell pool of each individual donor, we manually gated on FoxP3^+^ cells within proliferated CD4^+^ T cells and quantified the frequencies in all individual stimulated PBMCs. Similar to the viSNE result, a significant increase in Tregs amongst proliferated CD4^+^ T cells was observed in older adults (median of 19.80 vs. 5.27% in younger adults; *p* < 0.0001) (Figure 5A, gating strategy in Supplementary Figure S5). Importantly, increased frequencies of Tregs in the proliferated CD4^+^ T-cell population correlated with low CD4^+^ T-cell proliferative responses (*p* < 0.0001, *r* = −0.72) after AliB stimulation (Figure 5B). Further delineation of this Treg fraction showed no differences for individual CD25, TIGIT, Helios, and PD-1 marker expression between young and older adults (Supplementary Figure S6), whilst a significantly higher expression of CTLA-4 on proliferated Tregs (MFI 1627 vs. 1318 in younger adults; *p* = 0.0068) and a lower frequency of proliferated Tregs expressing CD127 (median of 4.44 vs. 7.77% in younger adults; *p* = 0.04) were observed in older adults (Supplementary Figure S6).

### Lower Levels of Active CD25^+^CD127^+^CD4^+^ T-Cells in Older Adults After AliB Stimulation Associated With Decreased CD4^+^ T-Cell Proliferation

Comparison of viSNE clusters also showed a significant decrease of cluster 6 in older adults compared to the younger age group (Figure 4E). Expression plots showed that cluster 6 contained CD4^+^ T cells with high expression of CD127 (Figure 4C). Further comparison of relative marker expression intensities between the identified clusters revealed that CD4^+^ T cells within cluster 6 also expressed relatively high CD25 and lower levels of inhibitory markers PD-1, CTLA-4 and TIGIT (Figure 4D). Quantification of manually gated CD25^+^CD127^+^CTLA-4^−^TIGIT^-^CD4^+^ T cells showed a similar decrease within the total proliferated CD4^+^ T cells of each individual at older age (median of 2.64 vs. 6.83% in younger adults; *p* = 0.008) (Figure 5C, Supplementary Figure S5). Moreover, decreased frequencies of these cells correlated with lower proliferative CD4^+^ T cell responses (*p* = 0.0094, *r* = 0.51) (Figure 5D).

## Discussion

Adults above 65 years of age become increasingly susceptible to pneumococcal infections associated with substantial morbidity and mortality. This could relate to immunosenescence of many components of the innate and adaptive immune system, including loss of effectivity of acquired specific immunity. Here, to our knowledge, for the first time evidence is presented for features of immunosenescence in CD4^+^ T cell responses to pneumococcal proteins that could contribute to susceptibility to pneumococcal infections at older age.

Two major observations were made in our study. First, we demonstrated that substrate-binding lipoproteins AmiA, AliA, AliB, AliC and AliD, are capable of inducing memory CD4^+^ T-cell responses in humans. Interestingly, despite the restricted expression of AliC and AliD to exclusively NESPs, we were able to detect substantial CD4^+^ T-cell responses against the two proteins. Both AliC and AliD share up to 60% sequence homology with AmiA, AliA and AliB (Alloing et al., 1994; Hathaway et al., 2004), with several conserved regions that could potentially harbour cross-reactive CD4^+^ T cell epitopes. Therefore, CD4^+^ T cells targeting these conserved regions might recognise pneumococci irrespective of their capsule status and contribute to protection against non-encapsulated strains that are on the rise due to the selective pressure mediated by current capsular polysaccharide-based vaccines (Bradshaw et al., 2018).

Second, and importantly, older adults showed lower proliferative CD4^+^ T-cell responses when stimulated with the immunodominant AliB protein compared to young adults, correlating with increased frequencies of proliferated Tregs and lower levels of an activated CD25^+^CD127^+^CTLA-4^−^TIGIT^-^CD4^+^ T-cell subset. Moreover, stimulation of PBMCs from older adults with AliB revealed decreased levels of pro-inflammatory cytokines IFNy and IL-17F and increased levels of IL-2.

Reduced CD4^+^ T-cell proliferation, which is a key feature of immunosenescence, was not associated with shifts in the CD4^+^ T-cell memory subsets in our aged cohort, similar to what others have shown (Czesnikiewicz-Guzik et al., 2008; Aiello et al., 2019; Pieren et al., 2019; Callender et al., 2020). This is in stark contrast with the human CD8^+^ T-cell compartment, known to undergo significant changes in Tnaïve, Tcm and Temra subset distribution with age (Czesnikiewicz-Guzik et al., 2008; Callender et al., 2020).

Interestingly, a previous study found low CD4^+^ T-cell proliferation in 2 years old children after *in vitro* challenge with detoxified pneumolysin, which progressively increased until the age of 20 years followed by a plateau until the age of 40 (Pido-Lopez et al., 2011). The findings on the memory CD4^+^ T-cell responses to AliB from our aged cohort shed light on the other end of the age spectrum, and together these studies may indicate an inversed relationship of CD4^+^ T-cell proliferation with IPD incidences throughout life. In addition, low specific CD4^+^ T-cell proliferation early in life is associated with positive carriage of *S. pneumoniae* (Zhang et al., 2007). Altogether, this suggests that the reduced pneumococcal-specific CD4^+^ T-cell proliferation observed in young children as well as older adults could play a role in the increased susceptibility to pneumococcal infections in these age groups.

Notably, our study indicated that low CD4^+^ T-cell proliferative responsiveness in older adults was associated with an increased frequency of proliferated Tregs and accompanied by significantly decreased levels of pro-inflammatory cytokines IFNy and IL-17F after AliB stimulation. Earlier studies on Tregs and cytokine effector functions of pneumococcal-specific CD4^+^ T cells in healthy younger individuals showed that both proliferation and production of IFNy and IL-17 cytokines are subjected to Treg-mediated suppression (Pido-Lopez et al., 2011; Mubarak et al., 2016). The combination of higher levels of responding Tregs and decreased inflammatory cytokines in our older cohort suggests that the CD4^+^ effector/Treg balance is tipped towards stronger regulation of pneumococcal-specific CD4^+^ T-cell response at older age. This is further supported by the comparable expression of markers classically associated with the suppressive function (FoxP3, TIGIT, Helios) on proliferated Tregs between young and older adults, suggesting that the Tregs were equally suppressive in both age groups, which is in line with findings in other studies (Gregg et al., 2005; Nishioka et al., 2006; Lages et al., 2008; Hwang et al., 2009). Moreover, increased Treg frequencies were associated with positive carriage of *S. pneumoniae* in children (Zhang et al., 2011). Based on this we hypothesize that the increased frequencies of specific Tregs seen at older age contribute to a less efficient CD4^+^ T-cell response against *S. pneumoniae* and thereby lead to increased susceptibility to colonization and subsequent development of IPD in the elderly population.

Interestingly, while expression of Treg-associated markers (FoxP3, TIGIT and Helios) did not differ between young and older individuals, we detected a decreased frequency of Tregs expressing CD127 (IL-7Rα) at older age. Low expression or absence of CD127 is considered as classic Treg characteristic. However, recent studies discovered that expression of CD127 on Tregs were beneficial for the development, survival and homeostasis of Tregs in *in vitro* and murine models (Mazzucchelli et al., 2008; Simonetta et al., 2010; Schmaler et al., 2015). To further clarify the role of decreased CD127 expression of Tregs in pneumococcal CD4^+^ T cell responses at older age, more in depth studies on this cell subset would be needed.

Tregs exert their suppression via several mechanisms including the secretion of IL-10 and cell-cell contact (Schmidt et al., 2012). Despite the increased frequency of Tregs in older adults, no difference was observed in produced IL-10 levels between younger and older adults. Instead, the Treg population in older adults expressed elevated levels of CTLA-4. This suggests that the Tregs in older adults may mediate their suppressive function mainly via cell-cell contact and other functions such as the secretion of TGF-β and IL-35 (Schmidt et al., 2012). This would need further research to be confirmed.

Another key finding of this study is the association between the low proliferative response in older adults and the reduced frequency of CD25^+^CD127^+^CTLA-4^−^TIGIT^-^ cells in the proliferating CD4^+^ T cell fraction after stimulation. While this subset of CD4^+^ T cells is relatively unknown, one study found that it predominantly consists of Th1, Th2, and to a lesser extent Th17 CD4^+^ T cells that actively proliferate and secrete cytokines upon polyclonal stimulation (Narsale et al., 2018). Decreased presence of CD25^+^CD127^+^CTLA-4^−^TIGIT^-^ CD4^+^ T cells could therefore contribute to the decreased levels of IFNy and IL-17F observed within our older age cohort. IFNy is essential for the protection against pathogens, as mice with non-functional IFNy or IFNy receptor were demonstrated to be extremely susceptible to microbial pathogens (Shtrichman and Samuel, 2001). IFNy polarizes macrophages towards a pro-inflammatory phenotype with opsonophagocytic properties (Herbst et al., 2011), whilst IL-17 is shown to be important in neutrophil and macrophage attraction and potentiation in pneumococcal immunity (Lu et al., 2008; Zhang et al., 2009; Wright et al., 2013). It would therefore be of interest to investigate if the decreased presence of CD25^+^CD127^+^CTLA-4^−^TIGIT^-^proliferated CD4^+^ T cells in older adults is causally related to the decreased levels of IFNy and IL-17F and could thereby result in less efficient clearance of *S. pneumoniae* via opsonophagocytosis by phagocytes.

IL-2 plays an essential role in promoting T cell proliferation (Pol et al., 2020). We detected significantly elevated levels of IL-2 in older adults, which is in striking contrast to the findings of other studies showing reduced IL-2 levels at older age (Whisler et al., 1996; Pieren et al., 2019). This may be explained by the difference in polyclonal versus antigen-specific stimulus between the studies that result in different kinetics of the response. Elevated levels of IL-2 might indicate less efficient IL-2 consumption by CD4^+^ T cells at older age and contribute to lowered proliferative response. Whilst no difference was detected in the expression of CD25 between the two age cohorts within our study, further studies on the expression of the other two IL-2 receptor subunits, CD122 (IL2R-β and IL15-Rβ) and CD132 (IL2R-γ) is required in order to fully assess the impact of age on the consumption of IL-2 by antigen-responding T cells.

We acknowledge that the age-related changes in CD4^+^ T-cell response observed in this study are limited to a certain class of pneumococcal proteins and serve as proof of concept. It remains to be determined whether the observed age-related changes can also be detected for other pneumococcal protein classes reported to induce CD4^+^ T-cell responses, such as choline-binding proteins and cytolysins (Zhang et al., 2007; Pido-Lopez et al., 2011; van de Garde et al., 2019). In addition, while antigen-specific proliferation of CD4^+^ T cells well exceeded background proliferation in our 7 days flow-based CellTrace diminution assay, the sensitivity and specificity of the assay may need further optimization to distinguish between specific and bystander activated CellTrace^dim^ CD4^+^ T cells. Finally, whilst CD4^+^ T cells are the main producers of the cytokines investigated in this study, it should also be noted that other cells within the PBMC are capable of secreting IFNy or IL-17F as well. Therefore, the age-related differences observed for these cytokines should not be solely attributed to immunosenescence of CD4^+^ T cells, but also to other innate and adaptive cell types as well.

Collectively, our data showed for the first time that this class of pneumococcal substrate-binding lipoproteins was able to induce CD4^+^ T-cell response in humans and that the pneumococcal-specific CD4^+^ T-cell immune response is altered in older adults. Excessive regulation by Tregs exerted on the pneumococcal-specific CD4^+^ T-cell immune response at older age may result in diminished inflammatory response and ineffective clearance of *S. pneumoniae*, which could lead to increased chances of developing IPD at older age.

Immunosenescence is not limited to CD4^+^ T cells, but has an impact on all aspects of the immune system. Considering that protection against *S. pneumoniae* also requires antibodies and the innate immune response (Zhang et al., 2009; Wright et al., 2013; Ramos-Sevillano et al., 2019), wide-scale immunological studies are needed to fully understand the impact of aging on innate and acquired pneumococcal-specific immunity that causes the enhanced susceptibility at older age.

## Data Availability

The raw data supporting the conclusion of this article will be made available by the authors, without undue reservation.
