# Supplementary material for: Diminished Pneumococcal-Specific CD4+ T-Cell Response is Associated With Increased Regulatory T Cells at Older Age
Source: Front Aging. 2021 Nov 3;2:746295. doi: 10.3389/fragi.2021.746295 (PMC9261371; doi:10.3389/fragi.2021.746295)
Supplement: Supplementary file 1 [file Table1.DOCX]

Supplementary Material

**
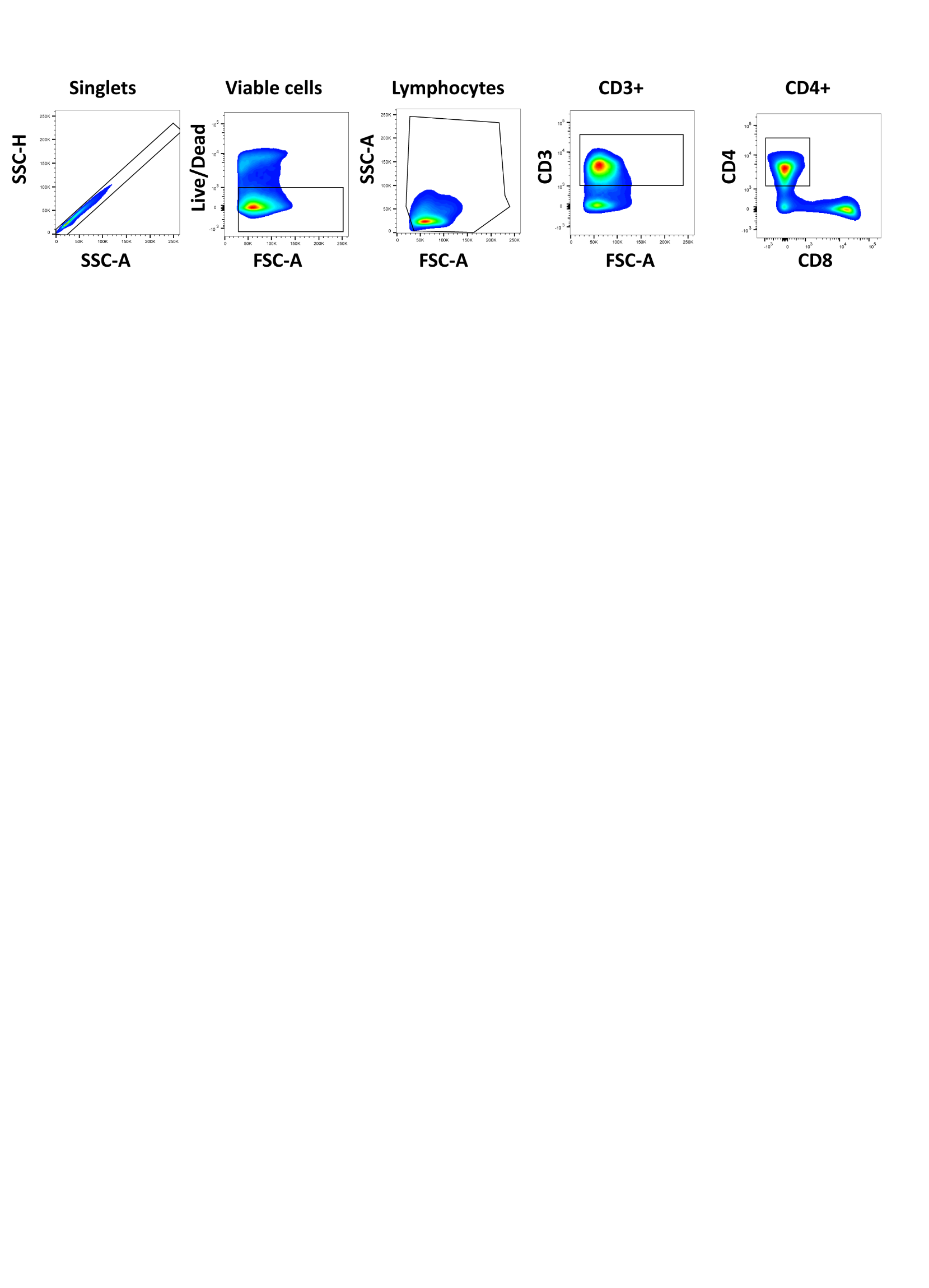
**

**Supplemental Figure 1. CD4^+^ T-cell gating strategy.** From left to right: plots show subsequent steps in the gating strategy for identification of singlets, viable cells, lymphocytes, CD3^+^ lymphocytes and CD4^+^ T cells, respectively, within individual ex vivo or cultured PBMC samples.

**
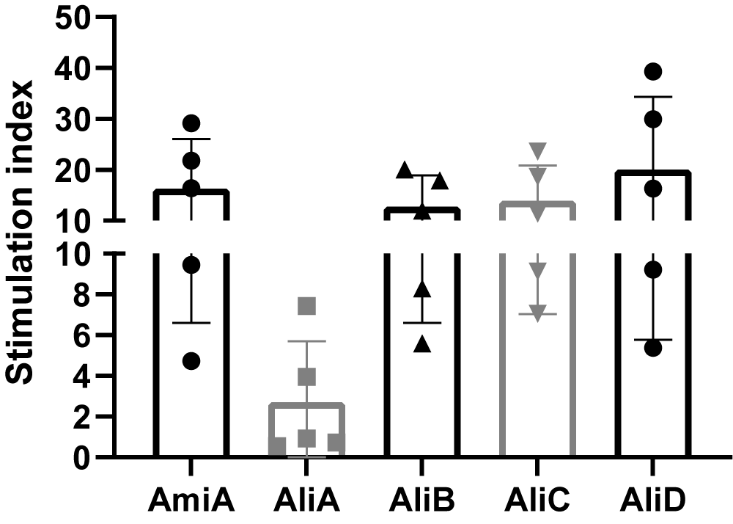
**

**Supplemental Figure 2. Immunogenicity screening of substrate-binding lipoproteins AmiA, AliA, AliB, AliC and AliD.** Proliferation of PBMCs (n= 5 donors) after stimulation with AmiA, AliA, AliB, AliC or AliD proteins for six days, as indicated. Stimulation indices represent fold tritium thymidine incorporation in protein-stimulated cultures relative to their respective medium controls. Bars represent the median with interquartile range of tested samples.

**
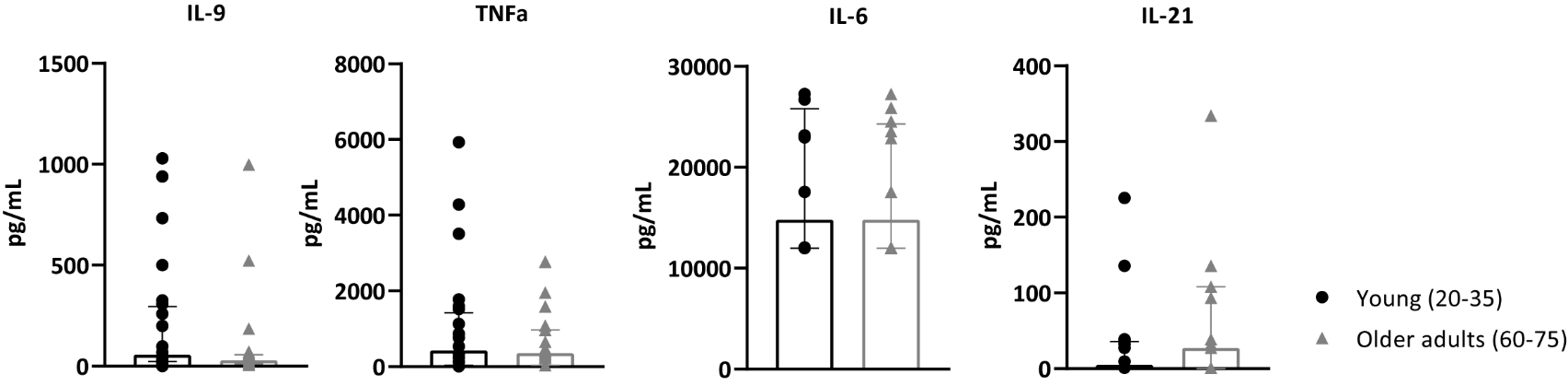
**

**Supplemental Figure 3. Cytokine levels in supernatants of young and older adults after AliB stimulation.** Bar graphs show levels (pg/mL) of IL-9, TNFα, IL-6 and IL-21 cytokines, as indicated, measured in the supernatant of PBMCs from young (n=12, black circles) and older (n=12, grey triangles) adults after seven-day AliB stimulation. Bars represent median with interquartile range per age group. Statistical significance is calculated with Mann Whitney *U*-test.

**
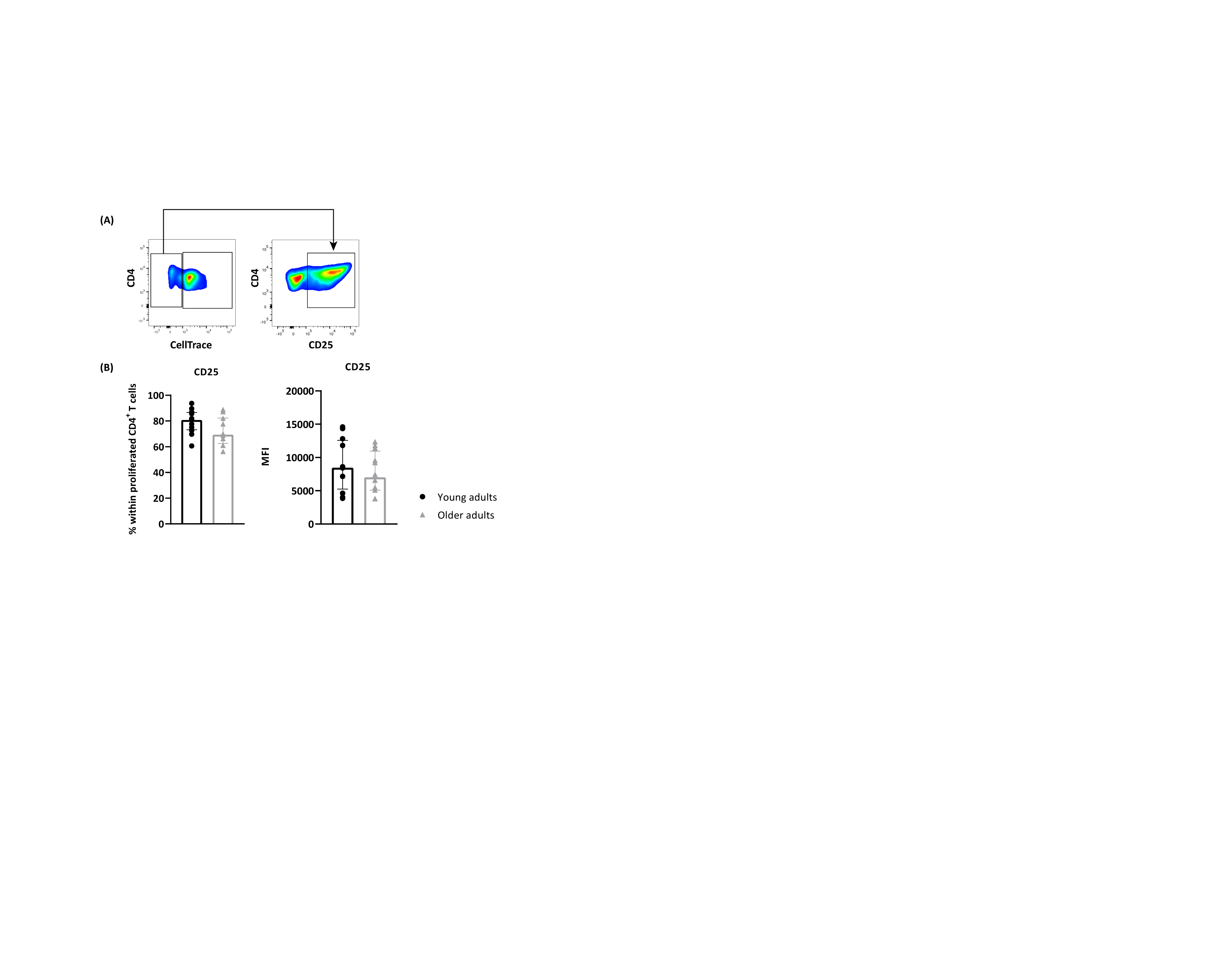
**

**Supplemental Figure 4. Frequency and expression of CD25 on proliferated CD4^+^ T cells from young and older adults after AliB stimulation.** CellTrace-labelled PBMCs from young (20-35 years, n=12, black circles) and older adults (60-75 years, n=12, grey triangles) were stimulated with AliB for seven days. **(A)** Gating strategy for CD25 expression on responding CD4^+^ T cells (CellTrace^dim^) (see general gating strategy for CD4^+^ T cells in Supplemental Figure 1) post AliB stimulation. **(B)** Bar graphs show frequencies of CD25 expression (left panel) and Median Fluorescence Intensity (MFI) (right panel) within proliferated CD4^+^ T cells per age group . Bars represent median with interquartile range. Statistical significance is calculated with Mann Whitney *U*-test (ns= not significant).

**
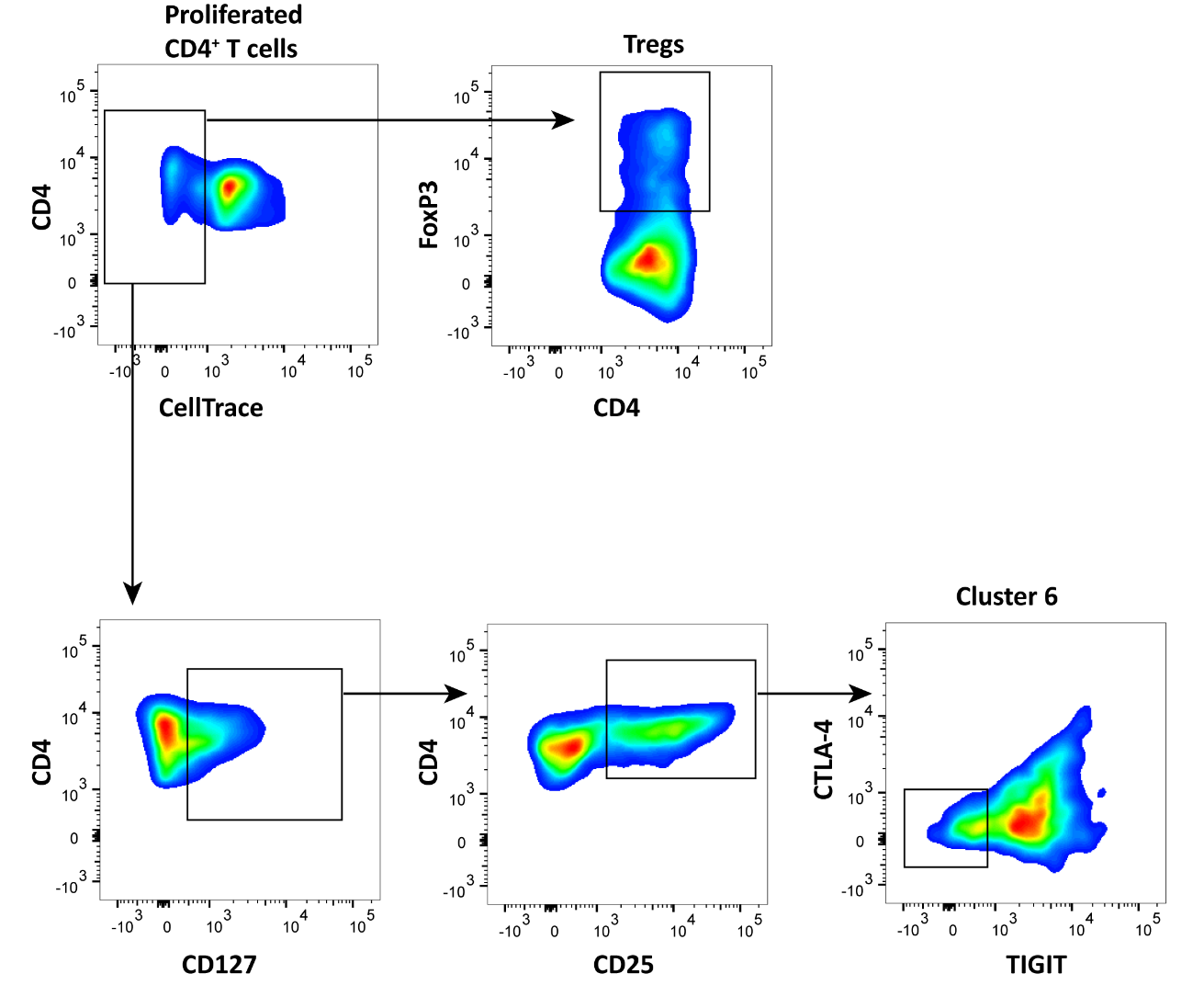
**

**Supplemental Figure 5. Gating strategy of identified viSNE clusters within proliferated CD4^+^ T-cells.** Plots show subsequent steps in the gating strategy of Tregs based on FoxP3 expression (upper graphs) and viSNE identified cluster 6 (based on Figure 4D ) (lower graphs) within the proliferated CD4^+^ T cells (see general gating strategy for CD4^+^ T cells in Supplemental Figure 1).

**
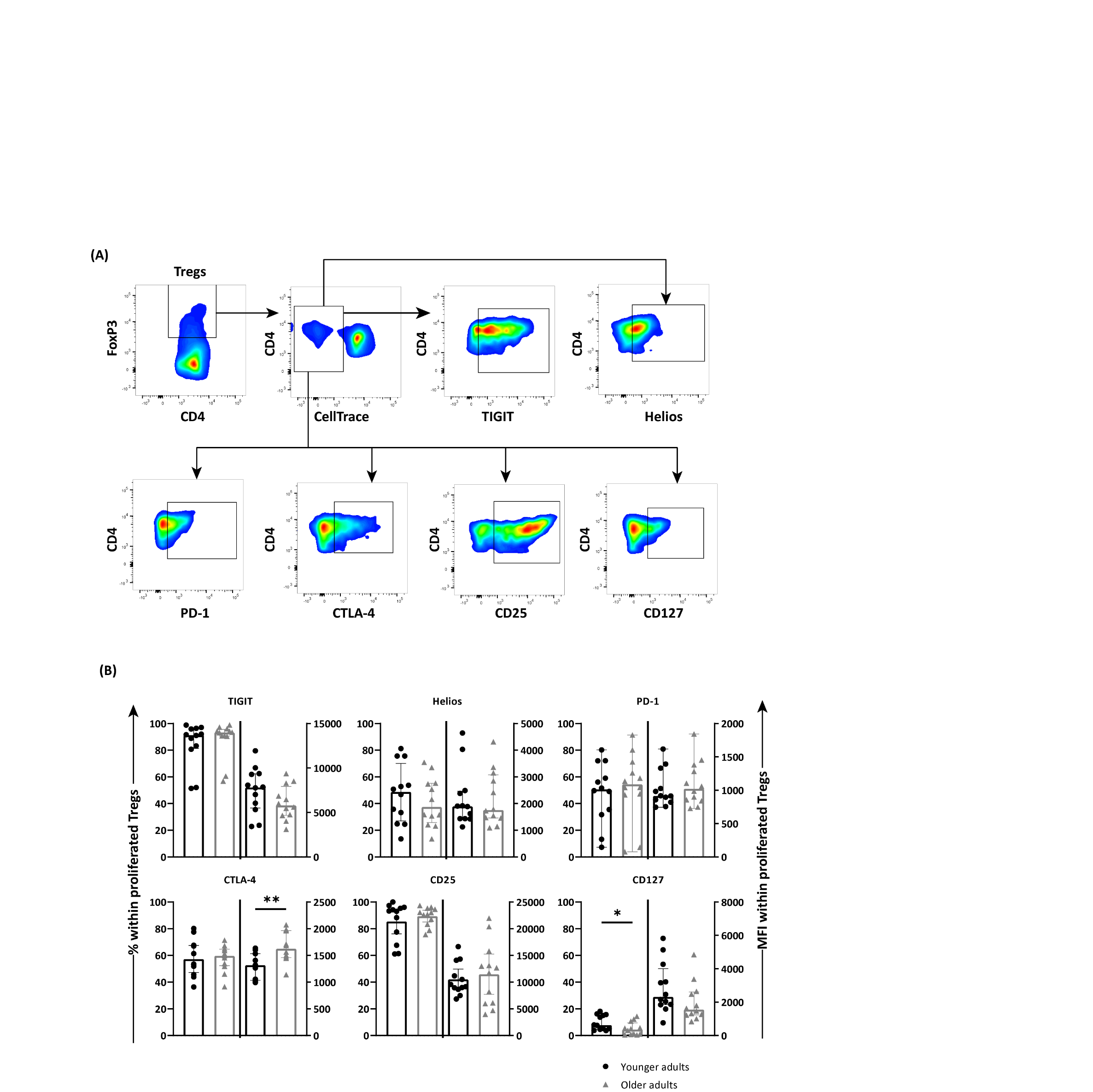
**

**Supplemental Figure 6. Marker expression on proliferated Treg populations in young and older adults. (A)** Gating strategy for identifying expression of TIGIT, Helios, PD-1, CTLA-4, CD25 and CD127, as indicated, on proliferated Tregs (CellTrace^dim^FoxP3^+^CD4^+^ T cells) after seven-day AliB stimulation (see general gating strategy for CD4^+^ T cells in Supplemental Figure 1). **(B)** Frequency (percentage, left Y-axis) and intensity (MFI, right Y-axis) of expression of TIGIT, Helios, PD-1, CTLA-4, CD25, or CD127, as indicated, on proliferated Tregs of young (n=12, black circles) and older (n=12, grey triangles) adults. Bars depict the median with interquartile range per age group. Statistical significance is calculated with Mann Whitney *U*-test (*= *p* <0.05, **= *p* <0.01).
